# A 90-Day Dietary Toxicity Study of Genetically Modified Rice T1C-1 Expressing Cry1C Protein in Sprague Dawley Rats

**DOI:** 10.1371/journal.pone.0052507

**Published:** 2012-12-27

**Authors:** Xueming Tang, Fangting Han, Kai Zhao, Yan Xu, Xiao Wu, Jinbin Wang, Lingxi Jiang, Wei Shi

**Affiliations:** 1 Biotechnology Research Institute, Shanghai Academy of Agricultural Sciences, Shanghai, People’s Republic of China; 2 Key Laboratory of Agricultural Genetics and Breeding, Shanghai Academy of Agricultural Sciences, Shanghai, People’s Republic of China; 3 College of Life and Environment Sciences, Shanghai Normal University, Shanghai, People’s Republic of China; Federal University of Pelotas, Brazil

## Abstract

In a 90-day study, Sprague Dawley rats were fed transgenic T1C-1 rice expressing Cry1C protein and were compared with rats fed non-transgenic parental rice Minghui 63 and rats fed a basal diet. No adverse effects on animal behavior or weight gain were observed during the study. Blood samples were collected and analyzed, and standard hematological and biochemical parameters were compared. A few of these parameters were found to be significantly different, but were within the normal reference intervals for rats of this breed and age, and were thus not considered to be treatment-related. Following sacrifice, a large number of organs were weighed, and macroscopic and histopathological examinations were performed with no changes reported. The aim of this study was to use a known animal model to determine the safety of the genetically modified (GM) rice T1C-1. The results showed no adverse or toxic effects due to T1C-1 rice when tested in this 90-day study.

## Introduction

Bt rice is modified to express the *cry* gene from *Bacillus thuringiensis* (Bt). It is resistant to Lepidoptera, Diptera, Coleoptera and Hymenoptera insects [1], [2]. Therefore Bt rice decreases yield losses of rice, the use of insecticides, levels of mycotoxins, and larval attacks [3], [4]. Bt crops including Bt corn, Bt cotton, Bt canola and Bt potatoes expressing *cry* genes are commercially grown in many parts of the world. Bt-transgenic crops were first grown commercially in 1996 [5], and since then the planting area of transgenic crops has increased steadily year by year. In 2011, the planting area of GM crops increased by 8%, reaching 160 million hectares [6]. Bt rice is not yet grown commercially despite extensive research to develop pest-resistant rice [7].

Cry proteins, which are large crystalline parasporal inclusions are produced by Bt during sporulation. Of the Cry proteins, the Cry1C protein encoded by the *cry1c* gene is highly toxic to approximately 35–40 insect species including stem borers, *Spodoptera exigua*, beet armyworm and the diamond back moth (*Plutella xylostella*) of lepidopteran pests [8]–[10]. The Cry1C protein can also be combined with *cry1A* and *cry1Aa* genes to develop two-toxin Bt crops, which can enhance the toxicity of *cry1C* against *Spodoptera exigua* and *Helicoverpa armigera* [11], [12].

Cry proteins show high species-specific toxicity against certain insects. The mode of action in the insect involves the toxin binding with specific receptors in the gut which is highly alkaline inducing osmotic imbalance, cell lysis and subsequent death of the insect [13]. The Cry proteins are regarded as harmless to mammals including humans, probably due to the acidified gut pepsinolysis and the lack of Cry protein binding sites on mammalian gut epithelial cells [14]. To date, no reported pathogenicity in mammals including humans has been caused by Cry proteins [15].

Although the Cry1C gene has been used to develop transgenic rice to control lepidopteran pests, the safety of transgenic rice in humans is unknown. Cao *et al.* observed that the Cry1C protein did not cause toxic effects in ICR (Institute of Cancer Research) mice following acute administration of the Cry1C protein at a high dosage of 5 g/kg body weight [16]. An acute oral toxicity test of CrylC protein was analyzed using denaturing gradient gel electrophoresis and the results showed that CrylC protein was safe in mice [17]. However, Bt rice T1C-1 expressing Cry1C protein has not been assessed for toxicity. To assess the safety of T1C-1 rice, toxicity assessments are essential.

In this study, 90-day feeding toxicity studies were conducted to assess the safety of T1C-1 in rats following sub-chronic exposure to 60% rice diets. The aim of this study was to determine Bt rice T1C-1 expressing Cry1C protein is a safe new source of food.

## Materials and Methods

### Test Materials

Bt rice T1C-1 and the corresponding parental rice Minghui 63 were obtained from Shanghai Agrobiological Gene Center. Seeds of T1C-1 and its parental line, Minghui 63 were produced in the season of 2011 in Hainan, China. The generation and selection of the transformed rice was described in the study by Wei *et al.* During multiplication of rice seeds, the performance of these seeds was consistent with previous observations. Leaf folders and stem borers did not damage the T1C-1 plants, while Minghui 63 was infested by both leaf folders and stem borers, leading to damaged leaves (caused by stem folders), dead hearts and white heads (caused by stem borers) in the field. All handling was reduced to maintain the freshness and quality of the rice grains [12].

### Characterization of Test Materials

Rice plants were generated by Agrobacterum-mediated transformation and positive transformants were selected on the basis of phosphotinothricin resistance. Transgene expression of Cry1C in mature seeds of rice T1C-1 was verified by immunological assay (Western blotting after analysis of total protein by SDS-PAGE) using rabbit polyclonal antibodies raised against Cry1C as the primary antibody, with HRP-conjugated goat anti-rabbit IgG as the secondary antibody. The protein was visualized using ECL detection as previously described [18].

### Animals and Housing

Sixty specific-pathogen-free Sprague Dawley (SD) rats (30 males and 30 females) were obtained from the Experimental Animal Centre of Fudan University (Shanghai, China). The rats were 6–7 weeks old at initiation of the tests. All animals were kept pair-wise in stainless steel wire cages at 22±1°C at a relatively humidity of 40–60%. Animal experiments and housing procedures were carried out in accordance with the laboratory animal administration rules of the Ministry of Science and Technology of the People’s Republic of China.

### Diet Formulation and Feeding

The purified or semi-synthetic rat diet used in the study was produced in-house based on the rodent diet AIN-93 [19]. The purified diet in the control group was based on cornstarch without rice. Both test diets contained 60% ground rice flour, either Minghui 63 rice or T1C-1 rice expressing Cry1C protein from the *cry1C* gene. Both diets were adjusted identically to ensure an adequate supply of macronutrients and vitamins after substitution with 60% rice, but no adjustments were made to outbalance the differences in the constitution of the rice (Diets compositions see table 1). The rats were allowed free access to both food and water.

**Table 1:** Composition of diets.

	T1C-1	Minghui 63	Control
T1C-1 Rice (g)	600.000	0	0
Minghui 63 rice (g)	0	600.000	0
Starch (g)	172.885	172.885	620.000
Sucrose (g)	32.625	32.625	117.000
Casein (g)	45.691	45.691	114.120
Soybean oil (g)	66.530	66.530	70.000
Cellulose acetate (g)	44.550	44.550	50.000
L- cystine (g)	3.000	3.000	3.000
TBHQ (g)	0.008	0.008	0.008
Choline chloride (g)	1.340	1.340	1.340
Ferric citrate (mg)	203.297	203.297	269.950
Copper carbonate (mg)	7.491	7.491	11.010
Manganese carbonate (mg)	9.071	9.071	20.920
Zinc carbonate (mg)	31.651	31.651	65.890
Calcium carbonate (g)	12.459	12.459	12.500
Magnesium oxide (mg)	502.741	502.741	840.000
Lemon rubber acid potassium (g).	1.673	1.673	2.450
Sodium chloride (g)	2.542	2.542	2.640
Potassium dihydrogen phosphate (g)	5.853	5.853	6.860
Potassium sulfate (g)	1.630	1.630	1.630
Potassium iodide (mg)	0.260	0.260	0.260
Sodium selenite (mg)	0.840	0.840	0.840
Ammonium molybdate (mg)	0.280	0.280	0.280
sodium metasilicate (mg)	50.610	50.610	50.610
chromic potassium sulfate (mg)	9.610	9.610	9.610
Lithium chloride (mg)	0.870	0.870	0.870
Boric acid (mg)	3.430	3.430	3.430
Sodium fluoride (mg)	2.210	2.210	2.210
Nickel sulfate (mg)	2.240	2.240	2.240
Vanadate amines(mg)	0.230	0.230	0.230
Vitamin B1 (mg)	5.173	5.173	6.000
Vitamin B2 (mg).	5.820	5.820	6.000
Vitamin B6 (mg)	6.362	6.362	7.000
Folic acid (mg)	1.787	1.787	2.000
D-biotin (UG)	182.512	182.512	200.000
Nick (mg)	18.805	18.805	30.000
Calcium pantothenate (mg)	15.000	15.000	15.000
Vitamin B12 (UG)	25.000	25.000	25.000
Vitamin E (FU)	75.000	75.000	75.000
Vitamin A (IU)	4000.000	4000.000	4000.000
Vitamin D3(IU)	1000.000	1000.000	1000.000
Vitamin K (UG)	900.000	900.000	900.000

### Experimental Design

Following one week of acclimatization, the rats were randomly divided into three groups, with 20 rats in each group (10 male and 10 female per treatment) according to mean body weight. The animals were observed twice daily, and both body weight and food consumption were measured twice a week. During the last week of treatment, blood samples were taken from the tail vein and collected in tubes coated with EDTA and heparin for hematology and biochemistry analysis, respectively. The animals were fasted overnight before sacrifice to minimize fluctuations in the parameters measured.

### Blood Biochemistry and Hematology

Alanine aminotransferase (ALT), aspartate aminotransferase (AST), total protein (TP), albumin (ALB), alkaline phosphatase (AKP), glucose (GLU), blood urea nitrogen (BUN), creatinine (CREA), calcium (Ca), phosphorus (P), cholesterol (CHOL), total glycosides (TG), high density lipoprotein cholesterol (HDLC), and low density lipoprotein cholesterol (LDLC) were evaluated. All analyses on blood plasma were performed on a Hitachi 7020 automatic biochemical analyzer (Hitachi, Tokyo, Japan).

The following hematology parameters were assessed using a Sysmex KX-21N Hematology Analyzer (Sysmex Corporation, Kobe, Japan): white blood cells (WBC), red blood cells (RBC), hemoglobin (HGB), hematocrit (HCT), mean corpuscular volume (MCV), mean corpuscular hemoglobin (MCH), mean corpuscular hemoglobin concentration (MCHC), platelets (PLT), lymphocyte percent (LYM%), lymphocyte absolute value (LYM#), red cell distribution width (RDW), platelet distribution width (PDW), mean platelet volume (MPV), and platelet larger cell ratio (PLCR%).

### Bacterial Counts

During the experimental period, fresh fecal samples were taken for microbial analysis from ten animals (5 males and 5 females) in each group by provoked defecation at day 30 and 60 of the experiment, and at termination of the study. Furthermore, terminal sacrifice samples from the ileum and duodenum were taken from the same ten animals in each group. The fecal and intestinal samples were treated as described by Poulsen et al. [20].

### Histopathology

A thorough necropsy was performed and the following organs were excised and weighed: liver, spleen, kidney, heart, lung, thymus, thyroid gland, muscle, small intestine, stomach, testis, and ovary. Paired organs were weighed as a total including left and right. These organs were immediately fixed in 4% buffered formaldehyde for histological processing. Tissue samples were embedded in paraffin, and 4 to 6 µm thick sections were then stained with standard hematoxylin-eosin for light microscopy. The main focus of the histopathological examination was the intestinal tract and related organs. From a total of 10 males and females per group the following tissues were selected for histological examination: heart, liver, kidney, skeletal muscle, spleen, stomach, small intestine, thyroid gland, thymus, ovary and testis.

### Statistical Analysis

Statistical comparisons were designed to determine whether the differences in the aforementioned response variables between groups were attributable to the Cry1C protein as compared to the control groups. Data obtained from the male and female Cry1C protein groups were compared separately using the values from the basal diet groups. Homogeneity of variance was analyzed by one way analysis of variance with the statistical software program, statistical product and service solutions (SPSS) 17.0 (SPSS Inc., Chicago, IL, USA). Differences were considered significant when p<0.05.

## Results

### Clinical Observation, Body Weight and Food Intake

**Figure 1:**
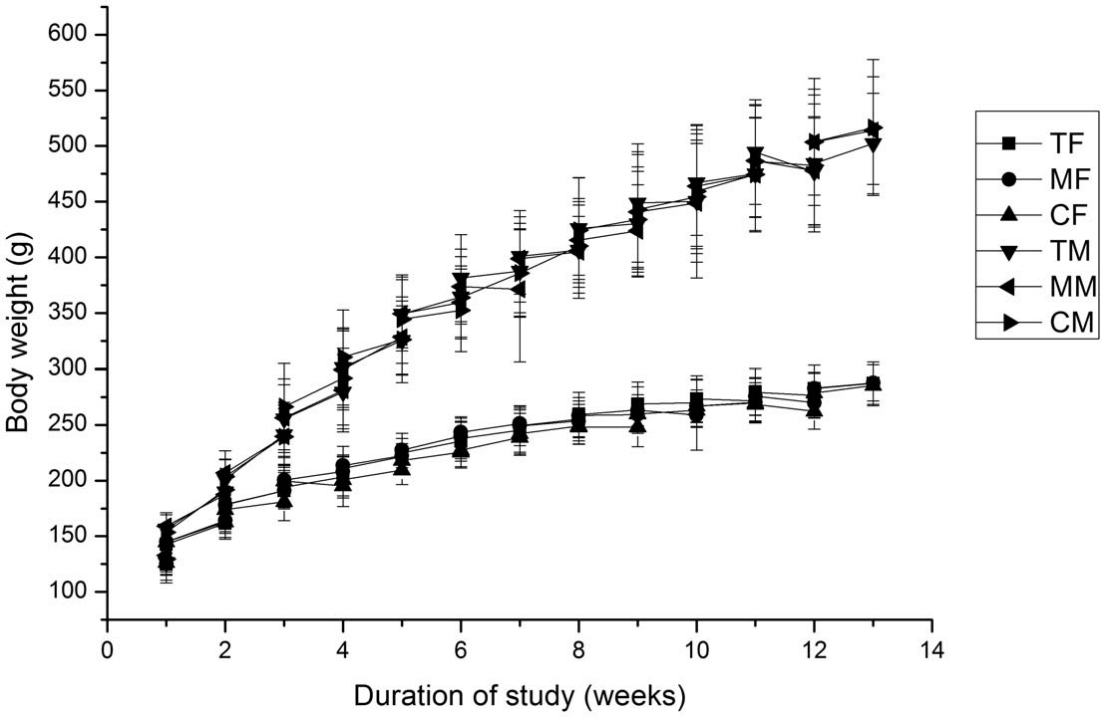
Mean live weight of rats fed with diets containing T1C-1,Minghui 63 rice. TF: T1C-1, Female. MF: Minghui 63, Female. CF: Control, Female. TM: T1C-1, Male. MM: Minghui 63, Male. CM: Control, Male.

No adverse effects on animal behavior were observed. Animal wellbeing was observed twice daily, and body weight and food consumption were measured twice a week. Growth curves for males and females are shown in Fig. 1 and illustrate normal and similar growth patterns in the three groups.

### Blood Biochemistry and Hematology

Results of the hematology and biochemistry assessments in males and females measured at study termination are listed in Table 2 and Table 3, respectively. There were no statistically significant differences in the three groups for most of the parameters measured. TP in females fed T1C-1 rice showed statistically significant differences as compared to control and Minghui 63 diets. CREA and CHOL in females fed T1C-1 rice showed statistically significant differences as compared to control and Minghui 63 diets, respectively. These results were not considered biologically significant as they were within the normal reference intervals [21].

**Table 2:** Hematology values in rats fed on transgenic rice T1C-1.

	T1C-1				Minghui 63			Control			
	female		male		female	male		female		male	
WBC (*10̂9/L)		8.11±0.86		12.35±1.15	10.40±0.78		12.76±1.05		8.78±0.44		9.09±1.20
RBC (*10̂12/L)		7.67±0.30		8.00±0.32	7.31±0.14		8.46±0.12		7.72±0.28		7.53±0.58
HGB (g/L)		164.60±6.32		162.00±5.79	155.00±2.21		168.70±3.80		166.30±6.42		154.00±11.79
HCT (%)		0.42±0.01		0.43±0.02	0.40±0.01		0.45±0.01		0.43±0.02		0.40±0.03
MCV (fL)		55.42±0.34		53.23±0.34	54.72±0.39		53.28±0.40		55.22±0.38		53.44±0.40
MCH (pg)		21.46±0.26		20.26±0.17	21.24±0.19		19.94±0.34		21.55±0.32		20.49±0.36
MCHC (g/L)		387.60±4.29		381.00±3.22	387.70±3.42		374.30±4.32		390.30±5.37		383.50±5.85
PLT (*10̂9/L)		843.80±66.80		906.30±42.32	836.30±52.00		821.90±24.53		744.80±56.74		812.10±83.42
LYM%		67.42±3.30		69.20±1.21	68.01±2.84		72.04±1.37		68.50±3.76		73.88±1.12
LYM# (*10̂9/L)		5.54±0.55		8.55±0.81	7.17±0.47		9.14±0.71		5.97±0.37		6.78±0.91
RDW (%)		29.96±0.32		32.72±0.34	29.57±0.39		31.91±0.26		29.91±0.27		32.59±0.36
PDW (fL)		12.51±0.57		12.78±0.66	12.58±0.64		13.12±0.54		11.86±0.43		11.67±0.20
MPV (fL)		8.78±0.12		8.95±0.18	8.74±0.15		8.90±0.14		8.53±0.10		8.53±0.11
PLCR%		0.18±0.01		0.19±0.02	0.19±0.01		0.20±0.01		0.17±0.01		0.16±0.01

The number of animals was 10 rats/sex/group; data is presented as group mean values±SD.

**Table 3:** Serum biochemistry in rats fed on transgenic rice T1C-1.

	T1C-1,		Minghui 63		Control		
	female,	male	female	, male	female		male
ALT (IU/L)	44.20±5.65	41.50±3.96	39.60±9.03	35.30±5.09	31.50±3.62	40.20±3.35	
AST(IU/L)	84.60±11.42	78.60±6.30	68.50±13.01	62.40±8.60	55.80±8.98	73.60±6.22	
TP (g/L)	65.97±2.17c	45.82±3.25	47.51±6.12	39.79±5.12	45.95±5.43	44.72±4.36	
ALB (g/L)	34.91±1.37	28.17±2.37	34.93±4.59	25.80±3.28	33.07±3.81	28.00±2.61	
AKP (IU/L)	125.30±13.31	288.60±21.26	115.90±11.44	268.60±38.89	124.20±1.87	267.80±24.36	
GLU (mM/L)	12.63±0.47	13.44±1.22	9.86±1.07	12.09±1.71	10.97±1.23	12.73±1.42	
BUN (mM/L)	9.38±0.41	8.12±0.54	7.83±0.87	7.78±0.97	7.80±0.70	8.28±0.70	
CREA(umol/L)	45.90±2.08a	39.60±2.34	36.80±3.35	32.40±3.18	35.90±3.78	37.90±3.42	
Ca (mM/L)	2.11±0.05	2.15±0.09	1.98±0.21	1.91±0.20	1.95±0.18	1.95±0.16	
P(mM/L)	1.41±0.08	1.84±0.09	1.28±0.12	1.54±0.19	1.27±0.14	1.67±0.14	
CHOL (mM/L)	1.82±0.13b	1.31±0.12	1.22±0.17	1.28±0.19	1.42±0.22	1.40±0.18	
TG (mM/L)	1.44±0.22	1.07±0.21	1.00±0.38	1.29±0.24	1.41±0.50	1.29±0.39	
HDLC (mM/L)	0.92±0.08	0.79±0.08	0.82±0.12	0.78±0.13	0.91±0.15	0.81±0.10	
LDLC (mM/L)	0.27±0.03	0.31±0.03	0.21±0.02	0.31±0.03	0.22±0.02	0.32±0.02	

The number of animals was 10 rats/sex/group; data is presented as group mean values±SD.

a P<0.05 between T1C-1 and control.

b P P<0.05 between T1C-1 and Minghui 63.

c P<0.05 in three groups.

### Microbiology

No significant differences in the bacterial microflora in the fecal samples were found in the three groups (data not shown). Significant microbiological findings in the small intestine are summarized in Table 4. Samples from the duodenum showed a 10% decrease in the Bifidobacterial population in the T1C-1 group compared to the control group (P<0.05).

**Table 4:** Bacterial counts in the small intestine of rats fed T1C-1 rice diet, Minghui 63 rice diet and basal diet.

	T1C-1	Minghui 63	Control
Bacterial counts in duodenum (log10 cfu/g intestinal content)			
Total aerobe	5.83±1.03 (9)	5.57±1.15 (9)	5.76±1.16 (9)
Total anaerobe	5.72±1.12 (8)	6.01±0.95 (9)	5.99±0.85 (9)
Lactobacili	6.10±0.72 (6)	6.45±0.98 (8)	6.39±1.12 (6)
Bifidobacteria	5.45±0.57 (6) *	6.18±0.66 (7)	6.23±0.67 (6)
Coliforms	3.01±0.13 (5)	3.32±0.25 (2)	3.39±0.17
Enterococci	4.01±1.33	4.03±0.98	4.01±1.29
Bacterial counts in ileum (log10 cfu/g intestinal content)			
Total aerobe	6.73±0.85 (8)	6.69±0.77 (8)	6.68±0.85 (9)
Total anaerobe	7.22±1.45	7.20±1.55	7.25±1.27
Lactobacili	7.02±1.09 (6)	7.13±1.21(6)	7.11±1.12
Bifidobacteria	5.69±0.43	5.74±0.22	5.70±0.54
Coliforms	5.51±0.34	5.37±0.23	5.49±0.19
Enterococci	5.93±1.22 (9)	5.97±1.03 (8)	5.97±1.23

Data are presented as group mean values±the standard error of the mean for ten animals; figures in baskets indicate the number of animals; * indicate significant difference in the bacteria counts in the three groups (P<0.05).

### Organ Weights

No significant differences in organ weights in the three groups were observed in this study. Details of organ weights are shown in Table 5.

**Table 5:** Absolute and relative organ weights for rats fed on T1C-1, Minghui 63 and basal diet.

	T1C-1			Minghui 63			Control		
	female		male	female		male	female		male
Absolute weight (g)									
spleen	0.534±0.02187	0.964±0.08935		0.554±0.04525	0.865±0.04256		0.538±0.03231	0.965±0.06517	
kidney	1.803±0.06055	3.287±0.12778		1.831±0.04159	3.442±0.15199		1.778±0.03519	3.397±0.11589	
liver	9.581±0.40898	16.507±1.12862		9.553±0.50285	18.494±0.84214		9.868±0.30409	17.548±0.81634	
Uterus and ovary (or testis)	1.231±0.16077	3.817±0.09824		1.081±0.10539	3.698±0.1118		1.089±0.0835	3.679±0.15647	
heart	1.062±0.04376	1.702±0.05645		1.049±0.02755	1.676±0.04764		1.065±0.02829	1.656±0.06943	
lung	1.573±0.05584	2.852±0.27756		1.648±0.06401	2.545±0.10027		1.631±0.04945	2.773±0.52406	
cerebrum	1.895±0.02994	2.174±0.024		1.954±0.02841	2.128±0.03021		1.903±0.02445	2.074±0.03403	
Relative weight (g/100g Body weight)									
spleen	0.19±0.019	0.2±0.068		0.19±0.049	0.16±0.027		0.19±0.029	0.18±0.033	
kidney	0.63±0.043	0.67±0.073		0.64±0.04	0.64±0.072		0.63±0.101	0.65±0.04	
liver	3.35±0.314	3.32±0.57		3.34±0.421	3.47±0.434		3.48±0.186	3.32±0.172	
Uterus and ovary (or testis)	0.44±0.197	0.78±0.079		0.38±0.118	0.7±0.093		0.38±0.079	0.7±0.086	
heart	0.37±0.036	0.35±0.037		0.37±0.028	0.32±0.032		0.38±0.034	0.31±0.026	
lung	0.55±0.05	0.59±0.231		0.58±0.045	0.48±0.059		0.58±0.051	0.54±0.369	
cerebrum	0.67±0.047	0.44±0.059		0.69±0.037	0.4±0.043		0.67±0.065	0.4±0.052	

### Gross Necropsy and Histopathology

**Figure 2:**
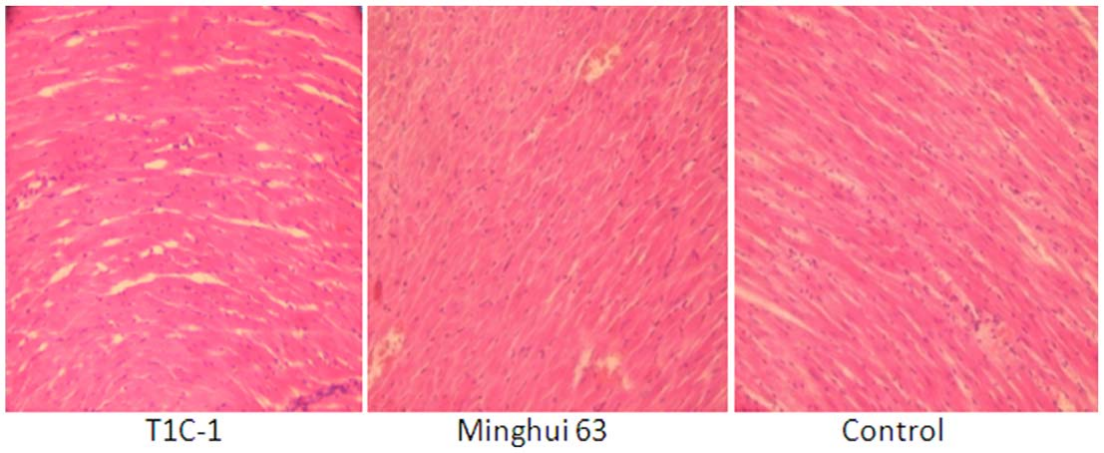
Heart tissue from rats fed with diets containing T1C-1,Minghui 63 rice. (H.E. 40×) For slitting line of heart, cross striation could be observed clearly. The nuclear was in the center, and cardiac muscle bleeding was not found.

**Figure 3:**
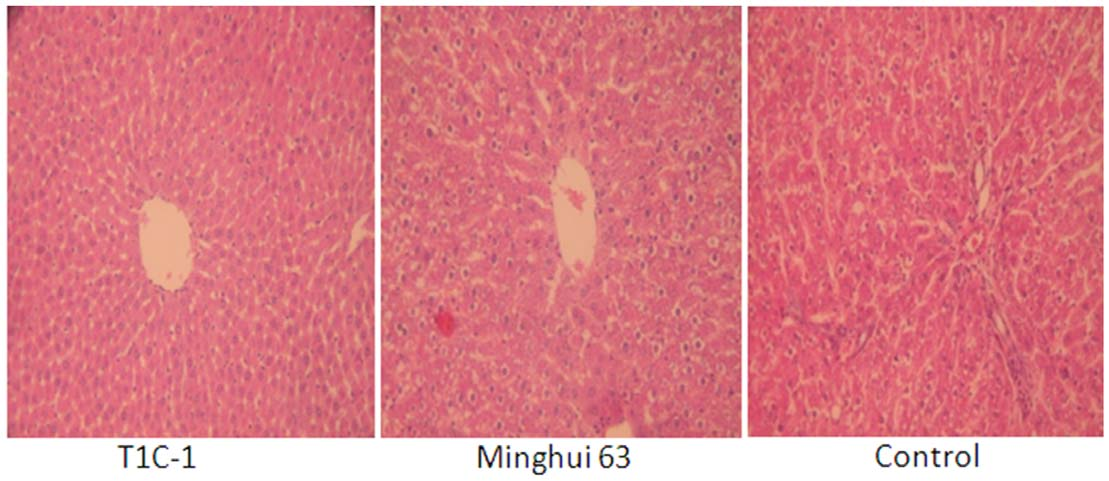
Liver tissue from rats fed with diets containing T1C-1,Minghui 63 rice. (H.E. 40×) Lobule structure and arrangement were in normal, with no liver cell edema, necrosis and degeneration.

**Figure 4:**
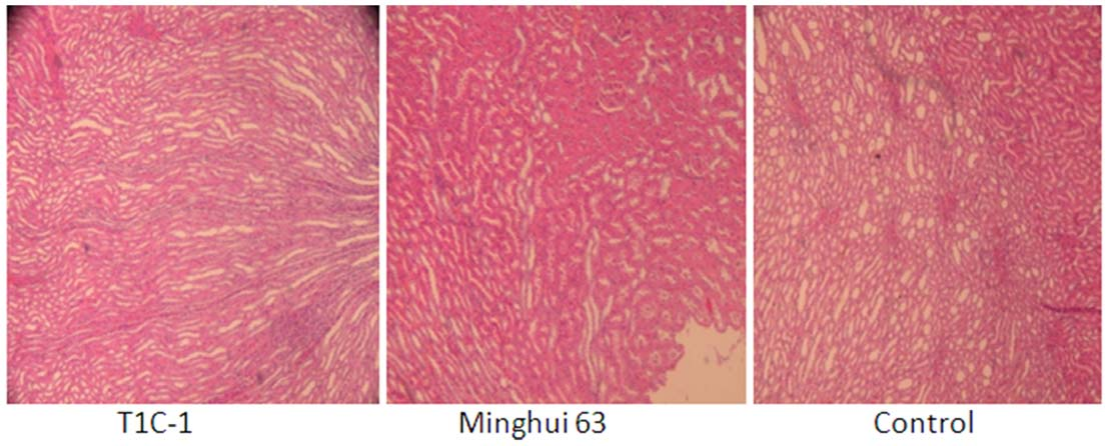
Kidney tissue from rats fed with diets containing T1C-1,Minghui 63 rice. (H.E. 40×) Glomerular and tubular structures around were complete and glomerular cysts was visible.

**Figure 5:**
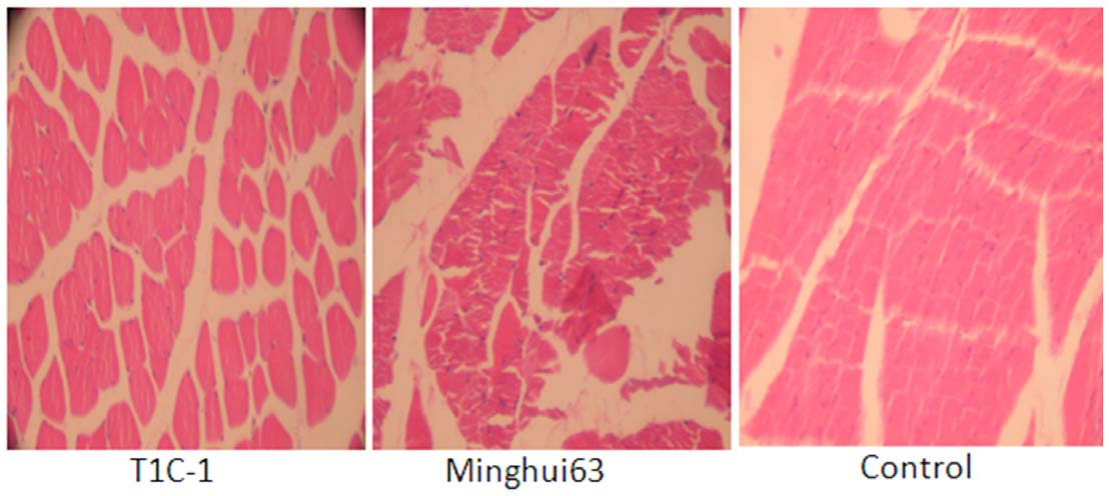
Skeletal muscle tissue from rats fed containing diets with T1C-1,Minghui 63 rice. (H.E. 40×) Muscle fibers arranged in neat rows with no abnormal structure.

**Figure 6:**
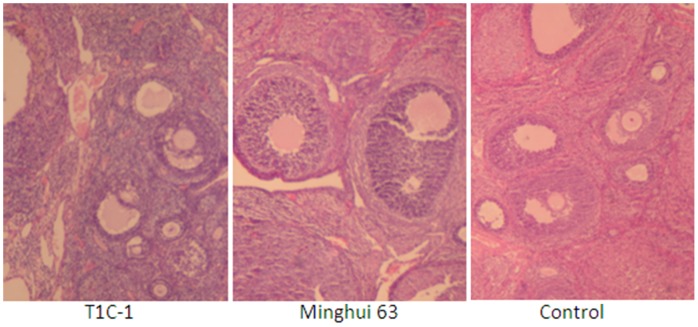
Ovary from rats fed with diets containing T1C-1,Minghui 63 rice. (H.E. 40×) Lesions were not observed in follicle and corpus luteum. Interstitial bleeding was not found.

**Figure 7:**
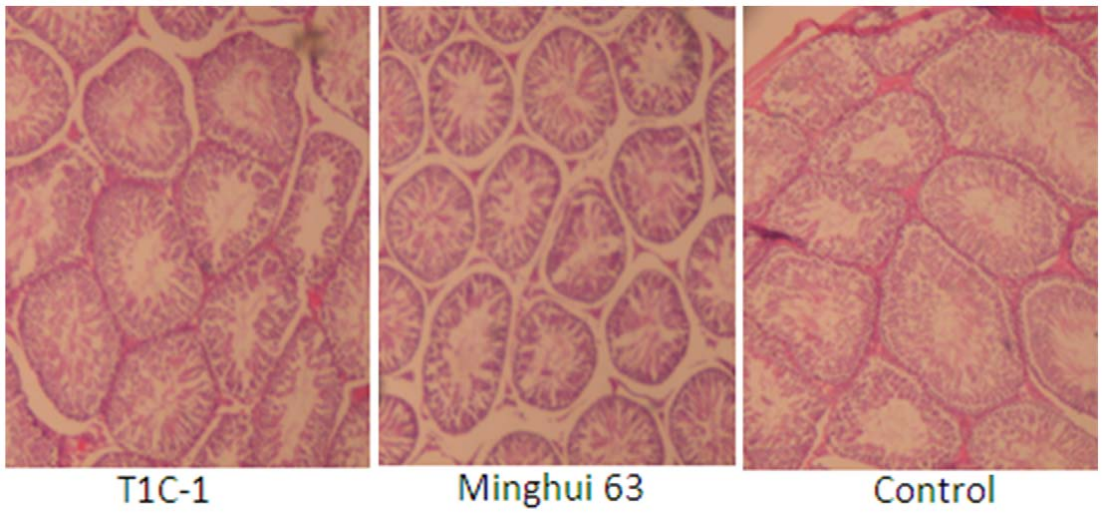
Testis from rats fed with diets containing T1C-1,Minghui 63 rice. (H.E. 40×) Structure of the seminiferous tubules and interstitial tissue were normal. No abnormal cell morphology were observed: spermatogenic cells in the seminiferous tubules arranged in layers at different developmental stages. Interstitial nucleus was large and spherical.

There were no gross pathological findings during necropsy and no group-related histopathologic observations were found. Histological examination for heart, liver, kidney, skeletal muscle, ovary and testis were as Fig. 2, 3, 4, 5, 6, 7.

## Discussion

Cry proteins have been used as pesticides for more than 40 years and numerous data from toxicity studies show no significant adverse effects of these Cry proteins on body weight or clinical observations. However, as genetically modified (GM) crops are becoming an increasing feature of agricultural landscapes, several international organizations have developed guidelines in attempt to guide the safety of GM foods or feeds for humans and livestock [22]–[29].

This article focused on the safety of *Bt* rice T1C-1 expressing Cry1C protein. The safety assessment of T1C-1 was based on scientific studies conducted with other GM *Bt* crops, and was consistent with the national standards of the People’s Republic of China for a new food resource.

Using compositional analysis, Wang *et al*. [30] reported that there were no statistically significant differences between the GM and parental rice. Schroder *et al.* attributed the significant differences between KMD1 and its parental rice Xiushui11 to biological variability rather than to genetic modification [14]. Therefore, it is possible that there are no statistically differences between T1C-1 and the parental rice Minghui 63 when analyzed using compositional analysis.

The concentration of Bt toxin in mature rice seeds is estimated to be 0.0165% of total soluble protein, which equates to approximately 15 mg toxin/kg rice. Rats fed with 60% rice diets in a 90-day assessment study was roughly equivalent to giving rats a mean daily dose of Bt toxin of 0.54 mg/kg body weight [14]. As 8400 mg Bt product/kg body weight/day in sub-chronic studies showed a no-observed-effect-level [13], possible toxicological findings with 0.54 mg kg/body weight/day would most likely derive from unintended changes introduced in the GM rice and not from the Bt toxin. Therefore, we designed our experiments so that the rats were fed on diets containing 60% T1C-1 or Minghui 63 rice.

Hematology analysis revealed that there were no significant differences in the three groups. Of the blood biochemistry parameters measured, TP, CREA and CHOL were found to be statistically different in the three groups. CREA and CHOL may be indicative of kidney and liver damage, respectively, but neither the differential count nor other significant findings on clinical observations, organ weights or pathology of kidney and liver supported this. In addition, with respect to blood biochemistry, the observed differences in TP, CREA and CHOL were all within normal reference intervals for rats of this breed and age. Therefore, the differences were considered insignificant.

The fecal samples obtained in this study did not reveal significant differences in bacterial counts for rats fed T1C-1 compared with the Minghui 63 and control groups. Minor effects on bacterial counts were observed in samples taken from the small intestine in the T1C-1 group. A study published in 1994 investigated bacterial changes in the rumen of cattle fed Bt corn, and it was found that Bt corn had no significant influence on the composition of the microbial population [31]. Schroder *et al*. reported that in their studies the amount of Bifidobacteria in the duodenum was reduced in the KMD1 group [14]. In the studies by Yuan *et al.*, rats were fed GM rice T2A-1 and no adverse effects were found when changes in specific fecal bacteria were monitored [32].

In this study, no pathological or histopathologic changes were found in rats fed a 60% T1C-1 diet. In a study by Schroder *et al.*, Cry 1Ab protein was not thought to be the reason for changes observed in the testis, which was supported by Wang *et al.* who carried out a comparable study on SD rats without any observed changes in the testis [14]. Brake *et al.* also proved that Bt corn had no apparent effects on the mouse reproductive system [33].

In the studies of Schardhein *et al*., the incidence of tumors in large series of albino rats ranging from 2 years of age and up has been reported as 42 to 59% in the Sprague-Dawley strain [34]. In our study, in order to rule out spontaneous factors to cause illnesses to ensure accuracy of the toxicity assessment for GM rice T1C-1, we chose 90 day as the study duration. To investigate the incidence of T1C-1 causing tumor, long term studies involving large number of rats will be required.

In this 90-day study, compared with the parental rice Minghui 63 and the control diet, the GM Bt rice T1C-1 exhibited no toxicological effects on SD rats when fed a 60% rice diet.
